# The immune microenvironment characterisation and dynamics in hormone receptor–positive breast cancer before and after neoadjuvant endocrine therapy

**DOI:** 10.1002/cam4.6425

**Published:** 2023-08-08

**Authors:** Gizem Oner, Glenn Broeckx, Christophe Van Berckelaer, Karen Zwaenepoel, Sevilay Altintas, Zafer Canturk, Wiebren Tjalma, Zwi Berneman, Marc Peeters, Patrick Pauwels, Peter A. van Dam

**Affiliations:** ^1^ Multidisciplinary Oncologic Centre Antwerp (MOCA) Antwerp University Hospital Edegem Belgium; ^2^ Center for Oncological Research (CORE) University of Antwerp Wilrijk Belgium; ^3^ Department of General Surgery Kocaeli University Kocaeli Turkey; ^4^ Department of Histopathology Antwerp University Hospital Edegem Belgium; ^5^ Department of Hematology Antwerp University Hospital Edegem Belgium

**Keywords:** biomarkers, breast cancer, endocrine therapy, hormone receptor, neoadjuvant, tumour‐infiltrating lymphocytes

## Abstract

**Background:**

Oestrogen receptor positive (ER+)/HER‐2 negative breast cancer (BC) is considered to be an immunologically cold tumour compared to triple negative breast cancer. Therefore, the tumour microenvironment (TME) of ER+/HER‐2 negative BC is understudied. The aim of this project is to investigate the TME and the immune response during neoadjuvant endocrine therapy (NET) and to correlate this with the treatment response in a real life setting.

**Methods:**

Expression of immune checkpoint receptors and immune cells was examined immunohistochemically, pre‐ and post‐NET in a cohort of 56 ER+/HER‐2 negative BC patients. They were treated with tamoxifen (*n* = 16), an aromatase inhibitor (*n* = 40) or a combination of an aromatase inhibitor with a PI3K inhibitor (*n* = 11) for a median duration of 6 months (range 1–32 months). Immunohistochemical staining with monoclonal antibodies for PDL‐1, PD‐1, TIM‐3, LAG‐3, CTLA‐4, CD4, CD68 and FOXP3 were performed. All staining procedures were done according to validated protocols, and scoring was done by a pathologist specialized in breast cancer. Positivity was defined as staining >1% on TILs. Response to NET was evaluated according to tumour size change on imaging and Ki‐67 change.

**Results:**

The median age was 61.02 (37–90) years. Diameter of tumour size decreased with a mean of 8.1 mm (−16 mm to 45 mm) (*p* < 0.001) during NET and the value of Ki‐67 value decreased with a median of 9 after NET (*p* < 0.001). An increase in PD‐L1 expression after NET showed a trend towards significant (*p* = 0.088) and CD‐4+ T cells significantly increased after NET (*p* = 0.03). A good response to NET defined as a decrease in tumour size and/or decrease of Ki‐67 was found to be associated with a longer duration of NET, a change of CD4+ T‐cells and a higher number of CD68+ tumour‐associated macrophages before the start of NET.

**Conclusion:**

The immune microenvironment plays an important role in ER+/HER‐2 negative BC. NET influences the composition and functional state of the infiltrating immune cells. Furthermore, changes in the immune microenvironment are also associated with treatment response.

## Abstract 2

The immune microenvironment also plays an important role in ER+/HER‐2 negative BC. A good response to NET defined as a decrease in tumour size and/or decrease of Ki‐67 was found to be associated with a longer duration of NET, a change of CD4+ T‐cells, and a higher number of CD68+ tumour‐associated macrophages before the start of NET. RCB is not effective in confirming long‐term prognosis for patients with HR (+) disease neither after NAC, nor after NET.

## 1 INTRODUCTION

The neoadjuvant approach has become one of the most useful strategies in breast cancer (BC) treatment, especially for human epidermal receptor‐2 (HER‐2) positive and triple‐negative breast cancer (TNBC) types. The use of neoadjuvant therapy not only offers clinical advantages such as lumen down staging and possible elimination of micrometastases, but also makes molecular and morphological analysis of the effects of the therapy possible. In addition, neoadjuvant therapy allows to evaluate the response to treatment and to evaluate the biological and molecular changes that may occur. The role of neoadjuvant chemotherapy (NAC) in localized breast cancer is well understood.^1^, ^2^, ^3^ Also, the interest in the clinical use of neoadjuvant endocrine therapy (NET) has increased recently. At the beginning, NET was preferred for the treatment of elderly patients with oestrogen receptor–positive (ER+) breast cancer who were not considered good candidates for surgery or systemic chemotherapy.^4^, ^5^ In the new European Society of Medical Oncology (ESMO) introduced during Covid‐19 period it is recommended to treat ER+ invasive breast cancer patients with NET.^6^ Furthermore, new treatments such as Phosphatidylinositol 3‐kinase (PI3K) or cyclin‐dependent kinase 4/6 (CDK4/6) inhibitors have begun to be combined with traditional endocrine drugs.^7^, ^8^, ^9^, ^10^

The tumour microenvironment (TME) can either promote or suppress tumour growth through different pathophysiological mechanisms. Tumour‐infiltrating lymphocytes (TILs) are the most important part of the TME and the presence of TILs in BC is related with a more favourable clinical outcome in more proliferative types of BC.^11^, ^12^, ^13^, ^14^ These studies have shown that high TILs infiltration is a good predictor of pathologic complete response (pCR) and a positive prognostic factor for HER‐2 positive BC and TNBC. In addition, tumour‐associated macrophages (TAMs) are also component of TME. TAMs can kill tumour cells or promote tumour cell growth and metastasis by changing their phenotype according to signals from the surrounding microenvironment.^15^, ^16^, ^17^ TAMs are abundant in BC and may form approximately 50% of the cell number in the tumour.^18^ However, the prognostic and predictive roles of TILs and TAMs in ER+ BC are controversial and little attention has been paid to the correlation between TILs, TAMs and response to NET.

Immune checkpoint molecules, including programmed cell death ligand 1 (PD‐L1, also known as B7‐H1 or CD274), programmed cell death protein 1 (PD‐1), lymphocyte activation gene‐3 (LAG‐3), cytotoxic T lymphocyte‐associated protein 4 (CTLA4) and T cell immunoglobulin‐3 (TIM‐3) are identified as crucial regulators of the immune response in TME^19^, ^20^, ^21^, ^22^ and takes part in changing the function of T cells. Tumour cells develop various strategies based on these molecules to escape immune surveillance. Immune checkpoint inhibitor therapy has appeared as an effective treatment method for many tumour types, including breast cancer, and has improved prognosis for many malignancies. Expression of immune checkpoint molecules is low in BC compared to other tumours such as non‐small cell lung cancer or melanoma. The expression of these molecules also varies with BC stage and molecular subtype, with the highest expression in TNBC followed by HER‐2 positive subtype.^11^ Although immune checkpoint ligands are less expressed in ER+ BC, it is not known how these markers affect TME in ER+ BC and how they are affected by hormone therapy. Therefore, a broader evaluation of TME in ER+/HER2‐ BC is required.

The goal of this paper was to investigate the TME and the immune response during NET and to correlate it to the treatment response in a real‐life setting. Another goal of this study was to elucidate how TME, and immune markers were altered during endocrine therapy in ER+/HER2‐ BC. Various immune markers were used to evaluate lymphocytes and macrophages in TME, and many aspects of tumour–immune interactions were investigated.

## 2 MATERIALS AND METHODS

### 2.1 Patient background

Between 2006 and 2018, 146 hormone receptor–positive (HR+) BC patients were treated with NET in both routine and clinical trial settings in the Multidisciplinary Breast Clinic of the Antwerp University Hospital. All patients (*n* = 56) who had HR (+), HER‐2 negative BC and had undergone surgery after NET were analysed in our study. Only 44 of these patients could be included in this study due to the fact that one of them had a pathological complete response and the tissue blocks of 11 patients had been exhausted. All patients had HR+ and HER2‐ BC and ER+/HER2‐ was divided into Luminal A like tumours and Luminal B like tumours and a Ki67 cut‐off (≥20%) was used to split these two subtypes.^23^ Oestrogen receptor (ER) and progesterone receptor (PR) expression was assessed using monoclonal antibodies EP1 (Ready‐to‐use (RTU) kit; Dako) and PR1294 (1:50; Dako) clones, respectively, and scored according to the Allred method. Allred scores of 3–8 were considered positive. Ki‐67 was stained using MIB‐1 (RTU kit Dako). Clinicopathological and follow‐up data of all patients were collected from hospital medical records. The absence of residual invasive carcinoma in the resected breast specimen and in all sampled regional lymph nodes after NET was defined as pCR.

### 2.2 Stromal tumour‐infiltrating lymphocytes (sTIL)

Morphological evaluation of TILs and TILs scoring was performed on haematoxylin and eosin (H&E) stained 4‐μm sections of formalin‐fixed paraffin‐embedded (FFPE) pretreatment tumour tissue and post‐treatment tumour tissue by different researchers according to the international consensus recommendations of the International TILs Working Group.^13^ All evaluations were performed avoiding areas with necrosis, technical artefacts and suboptimal tissue preservations. TILs were reported for the stromal compartment (% stromal TILs, sTIL) in all areas containing invasive tumour cells on the H&E slide. TILs were considered both as continuous variable and dichotomized in <10% (category 1), ≥10%–40% (category 2), and ≥ 40% (category 3).

### 2.3 Immunohistochemical analysis of immune checkpoint receptors

Immunohistochemical (IHC) staining was done in pretreatment and post‐treatment tumour tissue, and it was performed with an automatic Ventana BenchMark (Ventana Medical Systems, Tucson, AZ, USA) slide‐staining device. IHC staining of FFPE slides was performed using monoclonal antibodies against PDL‐1 (rabbit mAb; SP142 clone kit, Ventana Medical Systems, Tucson, AZ, USA) at 1: 100 dilution, PD‐1 (rabbit mAb; AC0255RUO, Epitomics, Abcam, UK) at 1:200 dilution, CTLA‐4 (rabbit policlonal Ab; PA5–23967, Thermo Fisher Scientific, UK) at 1:100 dilution, LAG‐3 (rabbit policlonal Ab D2G40TM; Cell Signalling Tech., MA, USA) at 1:200 dilution, TIM‐3 (XP rabbit, CST 45208S, Cell Signalling Tech., MA, USA) at 1:400 dilution, CD‐68 (mouse mAb; KP1, Dako, CA, USA) ready to use, CD‐4, mouse mAB clone SP35, Ventana, ready to use, OptiView detection method and FOXP3‐CD‐4 dual staining (FOXP3, mouse mAB clone 236 A/E7, Abcam, 1:200 dilution, Alkalic phosphatase detection method; CD4, mouse mAB clone SP35, Ventana, ready to use, OptiView detection method). Tonsil tissue was used as a control in the IHC procedure for all these antibodies.

Biomarkers were evaluated and reported following REMARK guidelines.^24^ Scoring for markers was done by measuring the percentage of cells stained in stromal tissue compartments. Positive staining was evaluated quantitatively, and thresholds used to collect data were chosen based on individual biomarker distribution and current standards. Scores of lymphocyte biomarkers were reported as absolute counts, and any positive expression (≥1% TILs per Tissue Microarray (TMA) core) was used for dichotomization into positive and negative cases.

### 2.4 Residual Cancer Burden Index

“MD Anderson Cancer Center Residual Cancer Burden Index” was also used to evaluate NET response. The two largest dimensions of the residual tumour bed (the largest tumour bed in multicentric cases is included in the calculation), the histologic assessment of the percentage of the tumour bed area that contains carcinoma, the histologic estimate of the percentage of the carcinoma in the tumour bed that is in situ, the number of metastatic lymph nodes and the diameter of the largest lymph node metastasis are required in order to calculate Residual Cancer Burden (RCB) index after NET treatment. The criteria's for calculating the RCB index were evaluated by the pathologists. RCB was determined using the official online RCB index calculator (http://www3.mdanderson.org/app/ medcalc/index.cfm?pagename = jsconvert3) and the RCB classification was based on this scoring.^25^ In this classification, the lowest category is considered as pCR (RCB‐pCR, similar to category RCB‐0), whereas the highest category (RCB‐III) is considered as neoadjuvant therapy resistant.

### 2.5 Statistical analysis

Data were analysed using R project in R studio (Version 3.6.3). Categorical variables were compared using Fisher's exact test or chi‐square test. ANOVA (continuous variables) and Pearson chi‐square test (categorical variables) were used to assess the relationship between the different parameters. Changes in quantitative biomarkers from before to after NET were made using Wilcoxon signed‐rank test. Significant parameters were included in a multivariate regression model. Survival data were last updated on September 1, 2021. All *p* values considered statistically significant when <0.05 and were calculated two‐sided.

## 3 RESULTS

### 3.1 Clinicopathological characteristics

Fifty‐six ER‐positive breast cancer patients [median age 61.02 years (37–90)] were enrolled in this study. Patient and tumour characteristics are presented in Table 1. Fifty‐six pretherapeutic core biopsies from patients with primary HR+ BC were eligible to be assessed for TILs. All these patients received NET and a majority of patients underwent breast‐conserving surgery (36/56, 64%) followed by radiotherapy (36/56, 64%). Median duration of NET was 6 months (1–37). Ten patients (18%) received an anthracycline and taxane based regimen postoperatively as adjuvant therapy. With a mean follow‐up of 47 months (10–119), two patients had a ‘breast cancer related’ event. One patient presented with only local recurrence, while local recurrence and metastasis (bone and liver metastases) were detected in the other patient 38 months after diagnosis. There were no breast cancer‐related deaths during follow‐up. Tumour tissues of 44 patients could be evaluated immunohistochemically before and after neoadjuvant endocrine therapy.

**TABLE 1:** Patient and tumour characteristics of the study population.

Patients characteristics (*N* = 56)		Before – NET *n* (%)	After – NET *n* (%)
Median age	61.02 years (37–90 years)		
Menopausal status	Premenopausal	16 (29)	
	Postmenopausal	40 (71)	
Tumour size (TNM – cT‐ ypT)	T0	‐	1 (2)
	T1	15 (27)	41 (73)
	T2	33 (59)	12 (21)
	T3	2 (4)	2 (4)
	T4	6 (11)	‐
Nodal status (TNM – cN‐ ypN)	N0	39 (70)	32 (57)
	N1	16 (28)	22 (39)
	N2	1 (2)	2 (4)
	N3	‐	‐
Intrinsic subtype	Luminal A	29 (54)	
	Luminal B	25 (46)	
Histology	Ductal	51 (91)	
	Lobular	5 (9)	
Nuclear Grade	G1	19 (34)	
	G2	25 (45)	
	G3	4 (7)	
	Unknown	8 (14)	
Neoadjuvant endocrine therapy	TAM ± LHRH	16 (28)	
	Aromatase inhibitors ± LHRH	40 (72)	
	+ PI3K blocker (trials)	11(20)	
Change of tumour size (median)	<= 4.5 mm		28 (50)
	> 4.5 mm		28 (50)
Change of Ki‐67 (median)	<= 9		26 (58)
	> 9		19 (42)
sTIL	<10% (category 1)	40 (71)	44 (80)
	≥10–40% (category 2)	13 (24)	9 (16)
	≥ 40% (category 3)	3 (5)	2 (4)
Residual Cancer Burden Category	RCB‐pCR		1 (2)
	RCB‐I		7 (12)
	RCB‐II		44 (79)
	RCB‐III		4 (7)

Abbreviations: LHRH, luteinizing hormone‐releasing hormone (LHRH); PI3K, phosphatidylinositol 3‐kinase (PI3K); RCB, Residual Cancer Burden; sTIL, tumour‐infiltrating lymphocytes; TAM, Tamoxifen; TNM, tumour node metastasis classification.

### 3.2 Immune biomarkers and changes in the immune microenvironment after NET

Median sTIL infiltration was 5% (range 1% to 85%) before NET and median sTIL infiltration was 5% (range 1%–80%) after NET (Spearman 0.46; *p* < 0.0001). sTIL infiltration decreased with a median of 1% (Q1: ‐ 2%‐ Q3: 4.5%) during NET with a trend towards significance (*p* = 0.08). Thirty‐three (58%) patients had a decrease of infiltrating sTIL and in four (7%) the number of sTIL remained stable.

**FIGURE 1:**
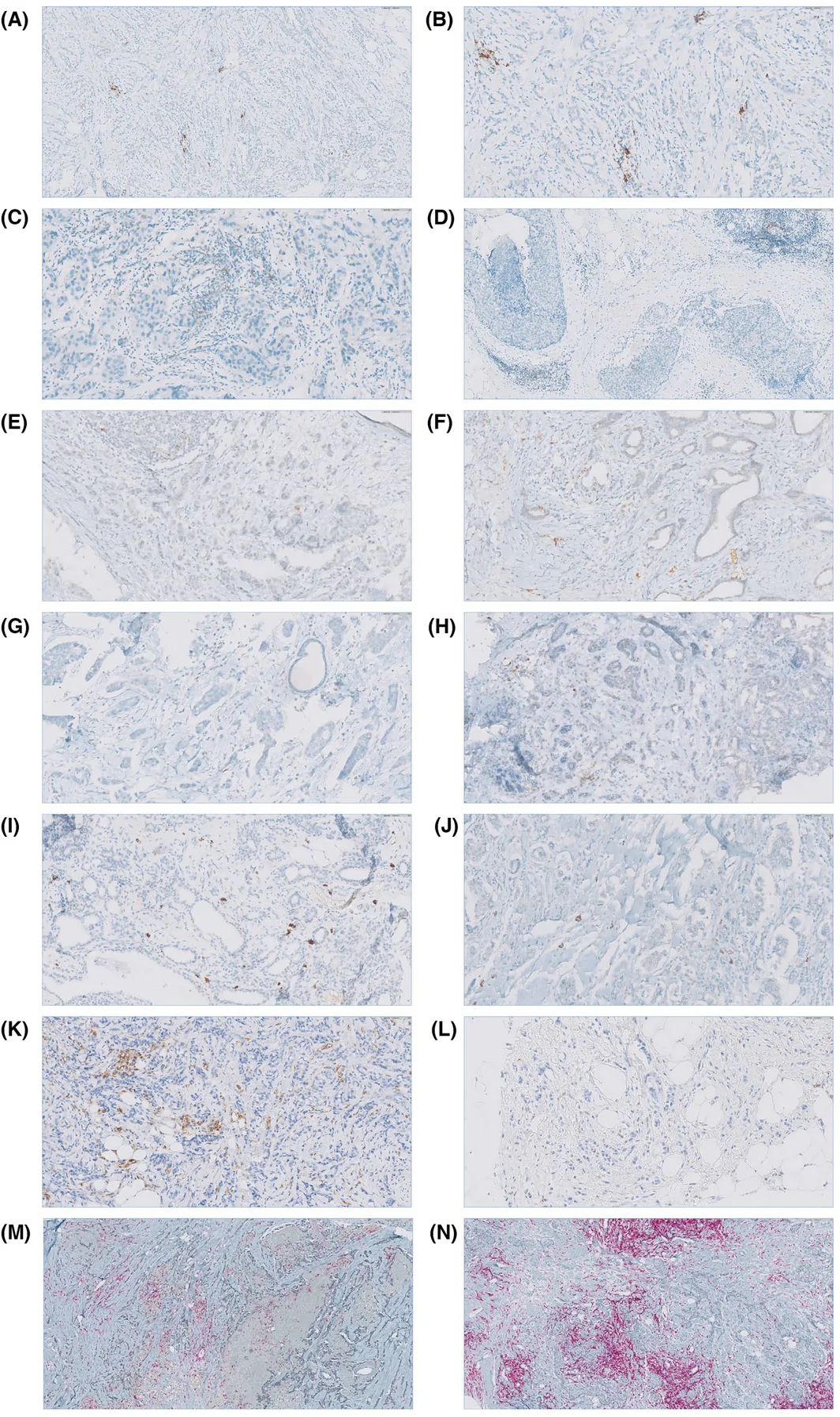
Immunohistochemical staining of FFPE slides was performed using monoclonal antibodies. Scoring for markers was done by measuring the percentage of cells stained in stromal tissue compartments. Positive staining was evaluated quantitatively, and thresholds used to collect data were chosen based on individual biomarker distribution and current standards. Scores of lymphocyte biomarkers were reported as absolute counts, and any positive expression (≥1% TILs per TMA core) was used for dichotomization into positive and negative cases. (A) PD‐L1 immunohistochemical expression is 5%–10% on the TILs (scale bar = 100 μm, ×20, HPF). (B) PD‐L1 immunohistochemical expression is 5%–10% on the TILs (scale bar = 40 μm, ×40, HPF). (C) PD‐1 immunohistochemical expression is 5%–10% on the TILs (scale bar = 40 μm, ×40, HPF). (D) PD‐1 immunohistochemical expression 5%–10% on the TILs (scale bar = 100 μm, ×20, HPF). (E) CTLA‐4 immunohistochemical expression 5%–10% on the TILs (scale bar = 40 μm, ×40, HPF). (F) CTLA‐4 immunohistochemical expression 10%–15% on the TILs (scale bar = 40 μm, ×40, HPF). (G) TIM‐3 immunohistochemical expression 10%–15% on the TILs (scale bar = 40 μm, ×40, HPF). (H) TIM‐3 immunohistochemical expression 1%–5% on the TILs (scale bar = 40 μm, ×40, HPF). (I) LAG‐3 immunohistochemical expression 10%–15% on the TILs (scale bar = 40 μm, ×40, HPF). (J) LAG‐3 immunohistochemical expression 5%–10% on the TILs (scale bar = 40 μm, ×40, HPF). (K) CD‐68 immunohistochemical expression 40% on the TILs (scale bar = 40 μm, ×40, HPF). (L) CD‐68 immunohistochemical expression 5% on the TILs (scale bar = 40 μm, ×40, HPF). (M) CD‐4 immunohistochemical expression 10% on the TILs and FOXP‐3 immunohistochemical expression 60% on the TILs (scale bar = 100 μm, ×20, HPF). N) CD‐4 immunohistochemical expression 70% on the TILs and FOXP‐3 immunohistochemical expression 30% on the TILs (scale bar = 100 μm, ×20, HPF). FFPE, formalin‐fixed paraffin‐embedded; HPF, high‐power field; TMA, tissue microarray.

**FIGURE 2:**
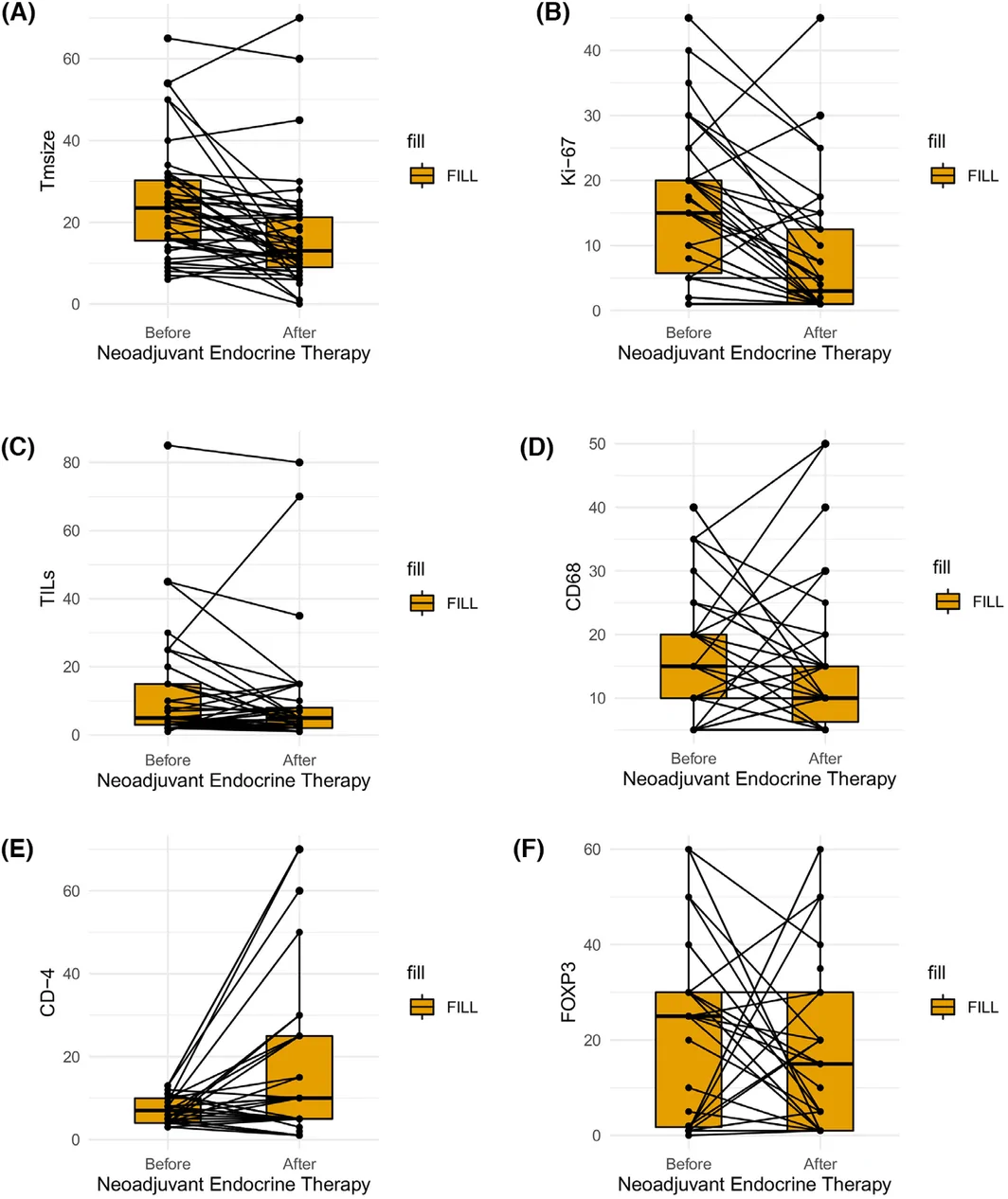
Boxplot graphs of the evolution of immune microenvironment before and after neoadjuvant endocrine therapy. Comparison of the immune markers before and after NET was done using Wilcoxon signed‐rank test. (A) Boxplot showing the evolution of tumour size during NET (*p* < 0.001), (B) Boxplot showing the evolution of Ki‐67 level during NET (*p* < 0.001), (C) Boxplot showing the evolution of sTIL during NET (*p* < 0.08), (D) Boxplot showing the evolution of CD‐68 expression level during NET (*p* < 0.11), (E) Boxplot showing the evolution of CD‐4 expression level during NET (*p* < 0.03), (F) Boxplot showing the evolution of FOXP3 expression level during NET (*p* < 0.14). Each boxplot represents the 25th–75th percentile with the median indicated as the central line and whiskers indicating 1.5 × interquartile range. Tm size, tumour size.

**FIGURE 3:**
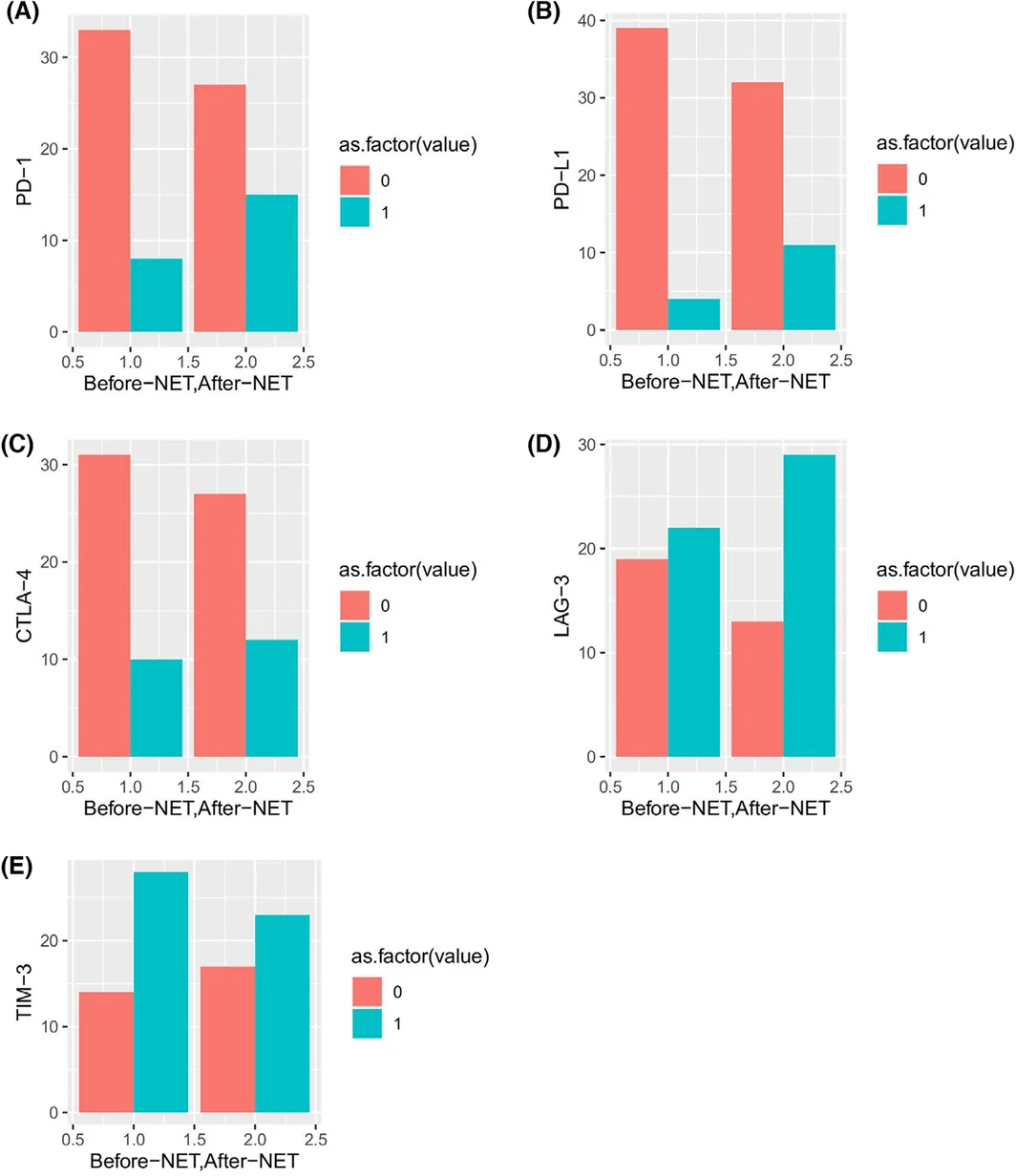
Bar graphs of the evolution of immune microenvironment before and after neoadjuvant endocrine therapy. Comparison of the immune markers before and after NET was done using chi‐square test. (A) The change in PD‐1 expression during NET (*p* < 0.16), (B) The change in PD‐L1 expression during NET (*p* < 0.088), (C) The change in CTLA‐4 expression during NET (*p* < 0.69), (D) The change in LAG‐3 expression during NET (*p* < 0.53), (E) The change in TIM‐3 expression during NET (*p* < 0.22). as.factor: 0 = defined as ≤1% for the immune markers, as.factor: 1 = defined as >1% for the immune markers.

The median values of pre‐NET staining percentages for CD‐68, CD‐4, and FOXP3 were 15% (5–40), 5% (1–13), and 22.5% (0–60), respectively, whereas after NET, the staining percentages of CD‐68, CD‐4 and FOXP3 were 10% (5–50), 10% (1–70) and 15% (1–60), respectively (Figure 1). CD‐4+ T cells significantly increased after NET (Wilcoxon signed‐rank *p* = 0.03). Most of the patients (91%) were negative (defined as ≤1%) for PD‐L1 before NET, and 75% of patients showed negative PD‐L1 expression after NET. While eight (18%) patients expressed PD‐1 (>1%) before NET, the number of patients expressing PD‐1 increased after NET (*n* = 15). CTLA‐4 expression was also negative in the majority of patients before and after NET (before NET: 77%, after NET: 73%). While 64% of the patients before NET showed TIM‐3 expression (>1%), 43% of the patients showed LAG‐3 expression (>1%) (Figure 1). The number of patients expressing both markers increased after NET, but this change was not statistically significant. Distribution of continuous and categorical parameters before and after NET is shown in Table 2 and Figures 2 and 3.

**TABLE 2:** Comparison of continuous and categorical parameters before and after NET. Comparison of the continuous parameters was done using Wilcoxon signed‐rank test. Comparison of the categorical parameters was done using a chi‐square test.

	Continuous parameters (median (min‐max))		
	Before NET	After NET	*p* Value
Tumour Size	23.5 (6–65)	13 (0–70)	**<0.001**
Ki‐67	15 (1–45)	3 (1–45)	**<0.001**
sTIL	5 (1–85)	5 (1–80)	0.08
CD‐68	15 (5–40)	10 (5–50)	0.11
CD‐4	5 (1–13)	10 (1–70)	**0.03**
FOXP3	22.5 (0–60)	15 (1–60)	0.14
FOXP3/CD‐4	2.307(0–15)	1 (0.03–12)	**0.04**

	Categorical parameters *n* (%)		*p* Value
	Before NET	After NET	
PD‐L1	4 (9)	11 (25)	0.088
PD‐1	8 (18)	15 (34)	0.16
CTLA‐4	10 (23)	12 (27)	0.69
TIM‐3	28 (64)	23 (52)	0.53
LAG‐3	19 (43)	29 (66)	0.22

Abbreviations: NET, neoadjuvant endocrine therapy; sTIL, stromal tumour‐infiltrating lymphocytes.

*Note*: Bold values denote statistical significance at the *p* < 0.05 level.

### 3.3 NET response

NET response assessed by tumour size change, Ki‐67 level change and RCB. Tumour size decreased overall with a median of 4.5 mm (Q1: 1%–Q3: 13.2%) (−16 mm–45 mm) (*p* < 0.001) during NET. While 45 patients (80%) responded well to NET, one patient had a stable tumour size, and 10 patients had an increased tumour size after NET treatment. The median Ki‐67 level was 15% before NET and the median Ki‐67 level was 3% after NET. Ki‐67 decreased with a median of 9.00 (Q1: 4.0%–Q3: 14.0%) and decrease in Ki‐67 value after NET was statistically significant (*p* < 0.001). For 36 patients the Ki‐67 levels decreased after NET, while Ki‐67 level of six patients increased after NET. The mean MD Anderson Cancer Center Residual Cancer Burden Index score was 2.02 (0–5.07) and NET response was moderate in 44 (79%, RCB‐II) patients. A pCR was achieved in only one patient and there was no significant association between pre‐to‐post‐NET change in biomarkers and RCB. In addition, there was no statistically significant difference between the NET response and the drugs used in the treatment of NET.

The tumour size change and the Ki‐67 value change were compared with the immune (checkpoint) parameters. (Tables 3 and 4). According to NET treatment response, patients were divided into two groups as good responders and bad responders. The patient group above the median value of tumour size change and Ki‐67 value change was defined as the group that responded well to the NET, while the patients below the median value were specified as the group that responded bad to treatment. The univariable model showed that tumour size change was strongly correlated with NET time (OR: 1.002, 95% CI: 1.005–1.025, *p* = 0.018, Table 4). Furthermore, tumour size change was significantly associated with CD‐68 expression before NET (OR: 1.016, 95% CI: 1.001–1.031, *p* = 0.03, Table 4), CD‐4+ T cells after NET (OR: 1.008, 95% CI: 1.000–1.016, *p* = 0.003 Table 4) and FOXP3/CD‐4 after NET (OR: 0.947, 95% CI: 0.901–0.997, *p* = 0.004, Table 4). On the contrary, there was no significant association of an immune (checkpoint) parameter with the Ki‐67 level decrease. A regression model with all significant immune parameters showed no significant relationship with decrease in tumour size.

**TABLE 3:** Tumour size change and Ki‐67 level change compared with categorical variables.

Parameter (*n* = 44)	Decreased tumour size relative to the median		*p* Value (chi‐square)	Decreased Ki‐67 level relative to the median		*p* Value (chi‐square)
	Good (*n*) (>4.5 mm)	Bad (*n*) (<=4.5 mm)		Good (*n*) (>9)	Bad (*n*) (<= 9)	
Menopausal status						
Premenopausal	4	6	0.71	3	5	0.42
Postmenopausal	18	16		16	10	
Intrinsic subtype						
Luminal A	10	10	1	13	15	0.0001
Luminal B	10	12		6	15	
Nuclear Grade						
G1	7	7	0.98	5	6	0.70
G2	10	11		11	7	
G3	1	1		1	1	
RCB class						
I	2	2	0.54	2	2	0.79
II	19	18		18	19	
III	2	1		2	1	
PD‐L1 before NET						
PD‐L1 (−)	19	20	1	17	14	0.60
PD‐L1 (+)	2	2		2	14	
PD‐L1 after NET						
PD‐L1 (−)	16	16	1	14	10	1
PD‐L1 (+)	5	6		5	4	
PD‐1 before NET						
PD‐1 (+)	18	15	0.28	12	13	0.17
PD‐1 (−)	2	6		6	1	
PD‐1 after NET						
PD‐1 (+)	15	12	0.28	13	6	0.18
PD‐1 (−)	5	10		5	8	
CTLA‐4 before NET						
CTLA‐4 (−)	17	14	0.31	15	8	0.34
CTLA‐4 (+)	3	7		3	5	
CTLA‐4 after NET						
CTLA‐4 (−)	13	14	0.81	12	10	1
CTLA‐4 (+)	7	5		6	4	
TIM‐3 before NET						
TIM‐3 (−)	8	6	0.74	7	5	1
TIM‐3 (+)	13	15		11	9	
TIM‐3 after NET						
TIM‐3 (−)	9	8	1	7	4	0.93
TIM‐3 (+)	11	12		11	9	
LAG‐3 before NET						
LAG‐3 (−)	12	7	0.27	5	8	0.23
LAG‐3 (+)	9	13		12	6	
LAG‐3 after NET						
LAG‐3 (−)	7	6	1	2	6	0.09
LAG‐3 (+)	14	15		16	8	

Abbreviations: RCB, Residual Cancer Burden; NET, neoadjuvant endocrine therapy.

*Note*: Bold values denote statistical significance at the *p* < 0.05 level.

**TABLE 4:** Univariate analysis examining tumour size change and Ki‐67 level change correlation with continuous variables.

Parameters	Tumour size change correlation				Ki‐67 level change correlation			
	Univariate analysis				Univariate analysis			
	OR	OR (95% CI)		*p* Value	OR	OR (95% CI)		*p* Value
		Lower	Upper			Lower	Upper	
Age	0.997	0.987	1.006	0.62	1.002	0.991	1.014	0.62
NET time	1.002	1.005	1.025	**0.018**	0.989	0.965	1.013	0.37
BMI	1.009	0.981	1.038	0.49	0.983	0.956	1.011	0.25
sTIL before NET	1.001	0.992	1.011	0.70	1.997	0.988	1.006	0.59
sTIL after NET	1.001	0.991	1.010	0.82	0.999	0.990	1.008	0.90
RCB index	0.994	0.833	1.186	0.95	0.092	0.896	1.331	0.38
CD‐68 before NET	1.016	1.001	1.031	**0.03**	0.998	0.980	1.016	0.84
CD‐68 after NET	1.011	0.998	1.025	0.100	1.006	0.992	1.021	0.35
CD‐4 before NET	1.001	0.965	1.039	0.93	1.000	0.961	1.042	0.96
FOXP3 before NET	1.002	0.993	1.011	0.60	1.0008	0.991	1.010	0.86
CD‐4 after NET	1.008	1.000	1.016	**0.03**	1.004	0.994	1.013	0.39
FOXP3 after NET	0.993	0.984	1.002	0.15	1.006	0.996	1.016	0.22
FOXP3/CD‐4 before NET	1.017	0.973	1.064	0.43	0.993	0.944	1.04	0.81
FOXP3/CD‐4 after NET	0.947	0.901	0.997	**0.04**	1.021	0.960	1.086	0.50
sTIL change	1.002	0.989	1.044	0.75	0.997	0.984	1.009	0.65
CD‐68 change	1.002	0.989	1.014	0.74	0.990	0.977	1.004	0.19
CD‐4 change	0.991	0.983	0.999	**0.05**	0.994	0.985	1.004	0.32
FOXP3 change	1.003	0.997	1.009	0.25	0.999	0.992	1.005	0.80
FOXP3/CD‐4 change	1.028	0.995	1.063	0.10	0.994	0.959	1.030	0.74

Abbreviations: BMI, body mass index; NET, neoadjuvant endocrine therapy; RCB, Residual Cancer Burden.

*Note*: Bold values denote statistical significance at the *p* < 0.05 level.

## 4 DISCUSSION

In this study, we explored the tumour microenvironment in HR (+), HER‐2 negative BC before and after NET. In addition, we sought to shed light on the actual impact of immune markers on the NET response. The study revealed that immunohistochemical profiles of HR (+), HER‐2 negative BC show significant variation before and after NET.

Nowadays NET is more frequently used in clinical practice. However, we have limited knowledge of how pathological stage, biomarker status and TME can be used to make decisions regarding other adjuvant therapies after NET. Treatment with NET affects sTIL concentration, and this paper may support the assumption that sTIL concentration decreases during NET. In this study, sTIL infiltration decreased by a median 1% during NET, with a trend towards significance (*p* = 0.08). Contrary to our result, Skriver SK. et al found an increase in stromal TILs during neoadjuvant letrozole treatment.^26^ The different results of studies in this area reveal the need for additional studies. HR+ breast cancer tends to have fewer immune infiltrates then HER‐2 positive and TNBC types and higher TILs were related with worse outcome in HR+ breast cancer.^27^, ^28^ Our study has not demonstrated a correlation between high sTILs and node‐positivity, grade, Luminal B subtype and younger age, but numbers are limited. Furthermore, sTILs were not correlated to Ki‐67 or response to NET in our study. Clinically, sTIL is considered as a positive prognostic factor in TNBC and HER‐2 positive BC.^27^, ^29^ On the contrary, sTIL was related with shorter overall survival (OS) in ER+/ HER‐2 negative cancer.^27^ As studies in ER+/ HER‐2 negative BC increase, we will gain a more precise understanding of the effects of TILs in this specific breast cancer.

Our results contribute to the literature by showing the biomarkers expressed in the TME in HR (+), HER‐2 negative BC and how the expression of these markers are affected after NET. In our research, immune checkpoint inhibitors showed lower expression percentages before NET treatment except for TIM‐3. Most of the studies on immune checkpoint inhibitors in ER+ BC have focused on PD‐L1 expression and its use in immune therapy.^30^, ^31^, ^32^, ^33^, ^34^ Recent literature stresses that ER+/HER‐2 negative BC had the lowest PD‐L1 expression and TIL density. In addition, PD‐L1‐positivity was found in 53.1% of ER+/HER‐2 negative BC.^30^ However, PD‐L1 positivity rate (9%) was found to be lower in our study. The expression of PD‐L1 has been shown to be a prognostic marker in BC, particularly for TNBC.^11^ Zerdes et al showed that the expression of PD‐L1 was also related with a favourable prognosis in early‐stage invasive ER+/HER‐2 negative BC.^31^ In this research showed that the expression levels of immune markers vary after NET. There are studies showing that oestrogens can affect immune cell and PD‐1/PD‐L1 expression in HR+ breast cancer cells.^35^, ^36^ Oestrogen is a potent stimulator of FoxP3+ regulatory T cell. The meta‐analysis study found that high FoxP3+ T cells were related with a worse prognosis for ER‐positive BC.^37^ Aromatase inhibitors (AI) also modulate TME and immune biomarker expression.^28^ After neoadjuvant AI therapy, HR+ BC showed a decrease in FOXP3+ and an increase in the CD‐8+/FOXP3+ T‐cell ratio.^28^ Our findings revealed a significant effect of NET treatment on CD‐4+ T cells and FOXP3+/CD‐4+ T‐cell ratio (*p* = 0.03 and *p* = 0.04, respectively). FOXP3+ T cell expression was decreased after NET, but this was not statistically significant. PD‐1, PD‐L1, CTLA‐4 and LAG‐3 expression increased after NET, but these increases were not statistically significant. Taken together, these findings demonstrate that antioestrogen therapy may downregulate some immune markers while enhancing other potential immunological targets, and NET therapy may be recommended as a supportive adjunct therapy in the future as a preparatory therapy to induce the antitumour immune response.

Macrophages constitute an important part of the TME of BC and there are few recent studies investigating the role of macrophages in HR+ breast cancer.^38^, ^39^, ^40^, ^41^, ^42^, ^43^, ^44^ Antibodies against CD‐68 and/or CD‐163 were used in these studies to detect macrophages in the TME. In our study, we preferred to use CD‐68 antibody, which is known as a pan‐macrophage marker. CD‐68 positive macrophage infiltration has been associated with poor prognostic BC features such as higher tumour grade, larger tumour size, lymph node metastasis, hormone receptor negativity, HER‐2 expression and an increase of Ki‐67 level.^38^, ^39^, ^40^, ^41^ Gwak et al. found that high infiltration of intratumoural, stromal and total CD‐68+ macrophages was associated with shorter disease‐free survival in HR+ BC.^40^ In our study, no correlation was demonstrated between CD‐68 positive macrophages and negative prognostic markers. By contrast, we found an association between higher CD‐68 levels pre‐NET and a bigger decrease in tumour size (*p* = 0.03, OR: 1.016, 95% CI: 1.001–1.031). The expression level of CD‐68 decreased after NET, but this was not statistically significant. The studies on the prognostic effect of CD68+ macrophage have different results. This inconsistency may be due to different methodologies such as quantification of stromal, intratumoural or total macrophages and different cut‐off points chosen to identify a high CD68+ macrophage infiltration during the evaluation of CD‐68 expression.^38^, ^39^, ^40^, ^41^, ^42^, ^43^, ^44^ In this study, only stromal CD‐68 expression was considered positive staining.

Response to neoadjuvant therapy is crucial to obtain predictive and prognostic information and RCB pathological scoring systems are used to assess residual disease. Previous research demonstrated that the prognostic impact of RCB on OS and recurrence‐free survival (RFS) are mainly driven by RCB class after NAC and a high RCB group has been related with a bad prognosis.^45^, ^46^, ^47^ However, RCB is not effective in confirming long‐term prognosis for patients with HR+ cancer neither after NAC, nor after NET.^47^, ^48^, ^49^, ^50^ The unfavourable impact of higher RCB scores on RFS and OS are not consistent on HR (+)/HER‐2 negative BC and luminal BC is shown less pCR. The studies have shown that pCR is less useful and also inaccurate as a marker of treatment response in NET and HR+ BC.^49^ In this study, although most of the patients (44/56) were in the RCB class II, a very low rate of local recurrence (2/56) and metastasis (1/56) were detected during follow‐up. pCR rates are low for ER+ tumours receiving NET therapy, but it is indicated that this rate can be increased with longer treatment time.^50^, ^51^ Because the response to endocrine therapy is slow, NET duration has ranged from 3 to 6 months in most clinical studies. There are studies showing that a longer NET duration can lead to better NET response rates.^49^ Llombart‐Cussac et al. showed a median time to maximum response with letrozole of 4.2 months.^52^ On the contrary, another study comparing NET durations of 4, 8 or 12 months showed a significant trend for higher pCR rates with longer NETs.^53^ There are also difficulties in evaluating the NET response. For this reason, it is fairly certain that a combination of physical examination, mammography, ultrasound scanning and/or MRI scanning are necessary for response assessment after NET.^4^ Our findings showed that tumour size decreased significantly after NET. Recent literature stresses the importance of Ki‐67 suppression rather than pCR in the evaluation of the effectiveness of NET and subsequent adjuvant chemotherapy decision.^54^, ^55^ This study reveals a significant effect of NET on Ki‐67 suppression. In our study, we could not show a clear correlation between Ki‐67 and TILs. However, in the literature different results have been presented regarding the correlation between Ki‐67 and TILs in ER+/HER‐2 negative BC.^27^, ^56^ As articulated by Fujimoto et al., high TILs were associated with better disease‐free survival in high Ki‐67, ER+/ HER‐2 negative BC.^55^ According to Denkert et al., high/low Ki‐67 did not affect the overall prognosis of ER+/ HER‐2 (−) breast cancers.^27^ In addition, this study showed that stratification by Ki‐67 did not alter the effects of TILs in luminal BC.^27^ The inconsistent results show that additional studies are crucial in this area.

The limitations of this exploratory research need to be acknowledged, most notably it is a retrospective study. Antihormonal NET treatment modalities were not uniform. Because of this, the sample group was heterogeneous, and the sample size was small to perform specific analysis for each subtype. In addition, the fact that our sample consisted only of HR (+), HER‐2 negative BC treated with NET limited the number of patients that could be included in our research. Also, NET studies are the lack of a validated method for a response evaluation and RCB index used in the assessment of NAC is not effective in the neoadjuvant endocrine setting.

## 5 CONCLUSION

We demonstrated that NET has an effect on ER+/HER‐2 negative BC microenvironment and the expression of biomarkers changes during the treatment. Furthermore, changes in the immune microenvironment are also associated with NET response. In addition, we highlighted the role of TAMs which play an important role in TME in HR+ BC. Further research is imperative to improve our understanding of the clinical usefulness of the biomarkers in TME, particularly in the ER+/HER‐2 negative BC subtype. A better understanding of the role of immune mechanisms in HR+ BC will be important in determining NET protocols and developing new treatment strategies in daily clinical practice in the future.

## AUTHOR CONTRIBUTIONS

**Gizem Oner:** Conceptualization (lead); data curation (lead); formal analysis (lead); funding acquisition (lead); investigation (lead); methodology (lead); project administration (lead); resources (lead); validation (lead); writing – original draft (lead); writing – review and editing (lead). **Glenn Broeckx:** Conceptualization (supporting); data curation (supporting); investigation (equal); methodology (supporting); resources (supporting); supervision (supporting); writing – original draft (supporting); writing – review and editing (supporting). **Christophe Van Berckelaer:** Conceptualization (supporting); formal analysis (supporting); methodology (supporting); software (supporting); validation (supporting); visualization (supporting); writing – review and editing (supporting). **Karen Zwaenepoel:** Data curation (supporting); formal analysis (supporting); investigation (supporting); methodology (supporting); supervision (supporting); validation (supporting); writing – review and editing (supporting). **Sevilay Altintas:** Supervision (supporting); visualization (supporting); writing – review and editing (supporting). **Zafer Canturk:** Investigation (supporting); resources (supporting); supervision (supporting); validation (supporting); visualization (supporting); writing – review and editing (supporting). **Wiebren Tjalma:** Resources (supporting); supervision (supporting); visualization (supporting); writing – review and editing (supporting). **Zwi Berneman:** Validation (supporting); visualization (supporting); writing – review and editing (supporting). **Marc Peeters:** Investigation (supporting); resources (supporting); supervision (supporting); validation (supporting); writing – review and editing (supporting). **Patrick Pauwels:** Conceptualization (supporting); investigation (supporting); resources (supporting); supervision (supporting); validation (supporting); visualization (supporting); writing – review and editing (supporting). **Peter A. Van Dam:** Conceptualization (supporting); data curation (supporting); formal analysis (supporting); investigation (lead); methodology (supporting); resources (supporting); supervision (lead); writing – original draft (supporting); writing – review and editing (supporting).

## FUNDING INFORMATION

This study is funded by UZA Foundation grant and Kocaeli University, Department of Scientific Research Projects with the following grant numbers ‘TSA‐2019‐1611’.

## CONFLICT OF INTEREST STATEMENT

The authors declare no conflict of interest.

## ETHICS STATEMENT

This study is conducted in accordance with the ethical standards of the University Hospital Antwerp (UZA) and received ethical approval from the ethics committee (File number: 20/26/348, Edge number: 001250).

## Data Availability

Data sharing is not applicable to this article as no new data were created or analysed in this study.
